# Transport anomalies and lattice effects in superconducting Nb-doped Bi$\vphantom{0}_2$Se$\vphantom{0}_3$

**DOI:** 10.1038/s41598-026-63608-7

**Published:** 2026-09-15

**Authors:** Yanan Li, Christian Parsons, Anand Prashant Dwivedi, William Nelson, Ryan Baumbach, Marvin A. Schofield, Arneil P. Reyes, Prasenjit Guptasarma

**Affiliations:** 1 Department of Physics, University of Wisconsin-Milwaukee, Milwaukee, Wisconsin 53211 USA; 2 National High Magnetic Field Laboratory, Tallahassee, Florida 32310 USA; 3 Florida State University, Tallahassee, Florida 32310 USA; 4 Present Address: College of Electronic and Information Engineering, Liaoning Technical University, Huludao, Liaoning 125105 China; 5 Present Address: Department of Physics, University of California Santa Cruz, Santa Cruz, California 95060 USA

**Keywords:** Materials science, Physics

## Abstract

The interplay between symmetry-breaking orders in topological materials dictates their exotic quantum states. Here, we report a combined transport, NMR, and electron diffraction study of Nb-doped Bi$\vphantom{0}_2$Se$\vphantom{0}_3$, with doping level *x* ($0 \le x \le 0.4$). Electron diffraction reveals superlattice features and periodic lattice distortions, accompanied by a pronounced resistivity anomaly between 150 and 200 K and NMR line broadening below this temperature, consistent with a temperature-driven lattice and electronic instability. The resistivity anomaly scales with nominal Nb concentration *x* and coexists with superconductivity at low temperatures. While its microscopic origin remains unresolved, our observations reveal competing possibilities, including charge ordering, structural disorder, and frozen lattice distortions, highlighting the rich interplay between structure and electronic states in this system. Our results position Nb$\vphantom{0}_x$Bi$\vphantom{0}_2$Se$\vphantom{0}_3$ as a tunable platform for investigating intertwined electronic and structural phenomena in topological materials.

## Introduction

The interplay between charge density wave (CDW) order and unconventional superconductivity is a central topic in condensed matter physics^1–4^, as both phenomena involve electron-phonon interactions and result in broken symmetries. This relationship is prominently represented in layered dichalcogenides^5–13^, where Fermi surface topology plays a critical role. Recently, the observation of symmetry breaking in Cu-, Nb-, and Sr-intercalated topological insulators like Bi$\vphantom{0}_2$Se$\vphantom{0}_3$ has spurred interest in their potential for topological superconductivity^14–19^. First-principles calculations and neutron diffraction studies propose that the unconventional superconductivity in these materials may be driven by a phonon singularity caused by strong Fermi surface nesting along the $\it \Gamma z$ direction^20,21^. Given the observed nesting and multiple Fermi pockets^22^, the emergence of a competing electronic order in intercalated Bi$\vphantom{0}_2$Se$\vphantom{0}_3$ systems, such as Nb$\vphantom{0}_x$Bi$\vphantom{0}_2$Se$\vphantom{0}_3$, is a compelling and expected possibility^23^.

As a member of the chalcogenide family, Bi$\vphantom{0}_2$Se$\vphantom{0}_3$ and its doped derivatives have shown potential for hosting charge ordering or charge density wave states in numerous experimental and theoretical studies^24–31^. Throughout this work, we use the term “charge ordering” to describe a real-space electronic modulation accompanied by a periodic lattice distortion and resistivity anomaly, without committing to the specific wave vector or long-range coherence implied by a canonical CDW. In the odd-parity superconductor Nb$\vphantom{0}_x$Bi$\vphantom{0}_2$Se$\vphantom{0}_3$^17^, symmetry-breaking features such as nematic order appear to coexist with superconductivity, persisting both below and above the superconducting transition temperature $T_c$^24^. This compound exhibits a complex electronic structure, featuring a Fermi pocket away from the center of the Brillouin zone in addition to the characteristic ellipsoidal Fermi surface^23^. The nesting expected between such multiple Fermi surfaces is a predicted driver of concomitant density waves in Nb$\vphantom{0}_x$Bi$\vphantom{0}_2$Se$\vphantom{0}_3$^15,23^. Furthermore, metal-intercalated Bi$\vphantom{0}_2$Se$\vphantom{0}_3$ and Bi$\vphantom{0}_2$Te$\vphantom{0}_2$Se have been experimentally shown to host charge-ordered-like states and superlattice structures^29–32^. While superlattice reflections have been observed in some doped Bi$\vphantom{0}_2$Se$\vphantom{0}_3$ systems^29,32^, a clear charge ordering transition, evidenced simultaneously by thermodynamic anomalies in transport, spectroscopic signatures of gap opening, and microscopic probes such as NMR, has remained elusive. This lack of comprehensive evidence has hampered a deeper understanding of the relationships between charge order, symmetry breaking, and superconductivity in these topological materials.

Given these expectations, we have systematically investigated Nb$\vphantom{0}_x$Bi$\vphantom{0}_2$Se$\vphantom{0}_3$ ($0 \le x \le 0.4$) using complementary probes. Our measurements reveal a pronounced electronic anomaly that shares key characteristics with charge-ordered systems: a resistivity anomaly, lattice distortions, and NMR line broadening. However, its microscopic origin remains unresolved. This anomaly emerges at elevated temperatures ($T_{\text {anom}} \sim 150$–200 K) and is followed by superconductivity at a much lower temperature ($T_c \sim 1.9$–3.5 K). Crystals were synthesized using a self-flux method followed by high-temperature quenching. Selected area electron diffraction reveals superlattice reflections and diffuse streaks, signaling periodic lattice distortions and fluctuations. Bulk transport measurements show a clear resistivity anomaly consistent with a temperature-driven electronic instability and likely partial gapping of the Fermi surface. Superconductivity is confirmed by a sharp drop in resistivity and diamagnetic response in DC magnetization. Furthermore, $\vphantom{0}^{209}$Bi NMR measurements on Nb$\vphantom{0}_{0.05}$Bi$\vphantom{0}_2$Se$\vphantom{0}_3$ provide microscopic evidence through homogeneous linewidth broadening below $T_{\text {anom}}$, indicative of an anomaly-driven redistribution of the electric field gradient. While the microscopic origin of the observed instability remains unresolved, we discuss several possible interpretations, including charge ordering, structural disorder, and frozen lattice distortions. Taken together, these observations position Nb$\vphantom{0}_x$Bi$\vphantom{0}_2$Se$\vphantom{0}_3$ as a platform to study the interplay between electronic instabilities, structural modifications, and topological superconductivity.

## Crystal structure and Nb doping

**Fig. 1:**
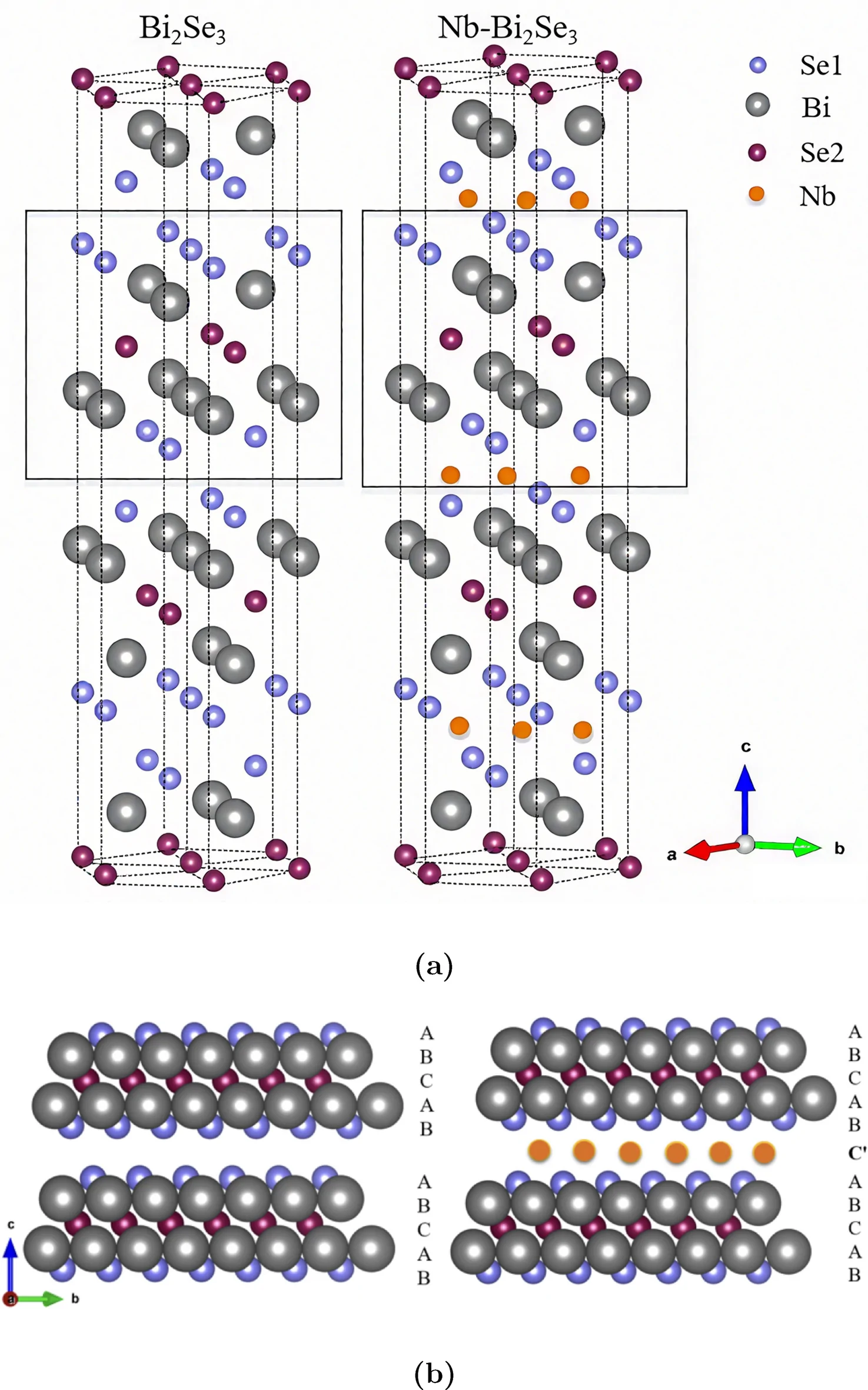
Crystal structure and symmetry breaking in Nb_x_Bi_2_Se_3_. (**a**) Comparison of the quintuple layer structure of Bi_2_Se_3_ (left) and the Nb-intercalated analogue (right). Atomic colors: Se1 (blue), Bi (grey), Se2 (red), Nb (orange). (**b**) The pristine *ABC* stacking order along the *c*-axis (left) is disrupted by Nb intercalation into the van der Waals gap (right). The Nb atom (C’) breaks the original periodicity, altering the stacking sequence from the ideal-A-B-C-pattern.

Figure 1(a) compares the crystal structures of pristine Bi$\vphantom{0}_2$Se$\vphantom{0}_3$ and Nb-intercalated Bi$\vphantom{0}_2$Se$\vphantom{0}_3$. Pristine Bi$\vphantom{0}_2$Se$\vphantom{0}_3$ (left panel of Fig. 1(a)) crystallizes in a rhombohedral structure with the space group $D_{3d}^{5}$ ($R\bar{3}m$)^33^. Its structure comprises quintuple layers (QLs) with a -Se1-Bi-Se2-Bi-Se1- sequence, which stack along the trigonal *c*-axis. The bonding within each QL is strong and covalent, whereas adjacent QLs are bound by weak van der Waals forces, facilitating easy cleavage of the crystal. As illustrated in the left panel of Fig. 1(b), the atomic layers adopt an ABC stacking sequence: -A(Se1)-B(Bi)-C(Se2)-A(Bi)-B(Se1)-, where the Se1 atoms terminate the QL surfaces and Se2 atoms occupy the central layer.

**Fig. 2:**
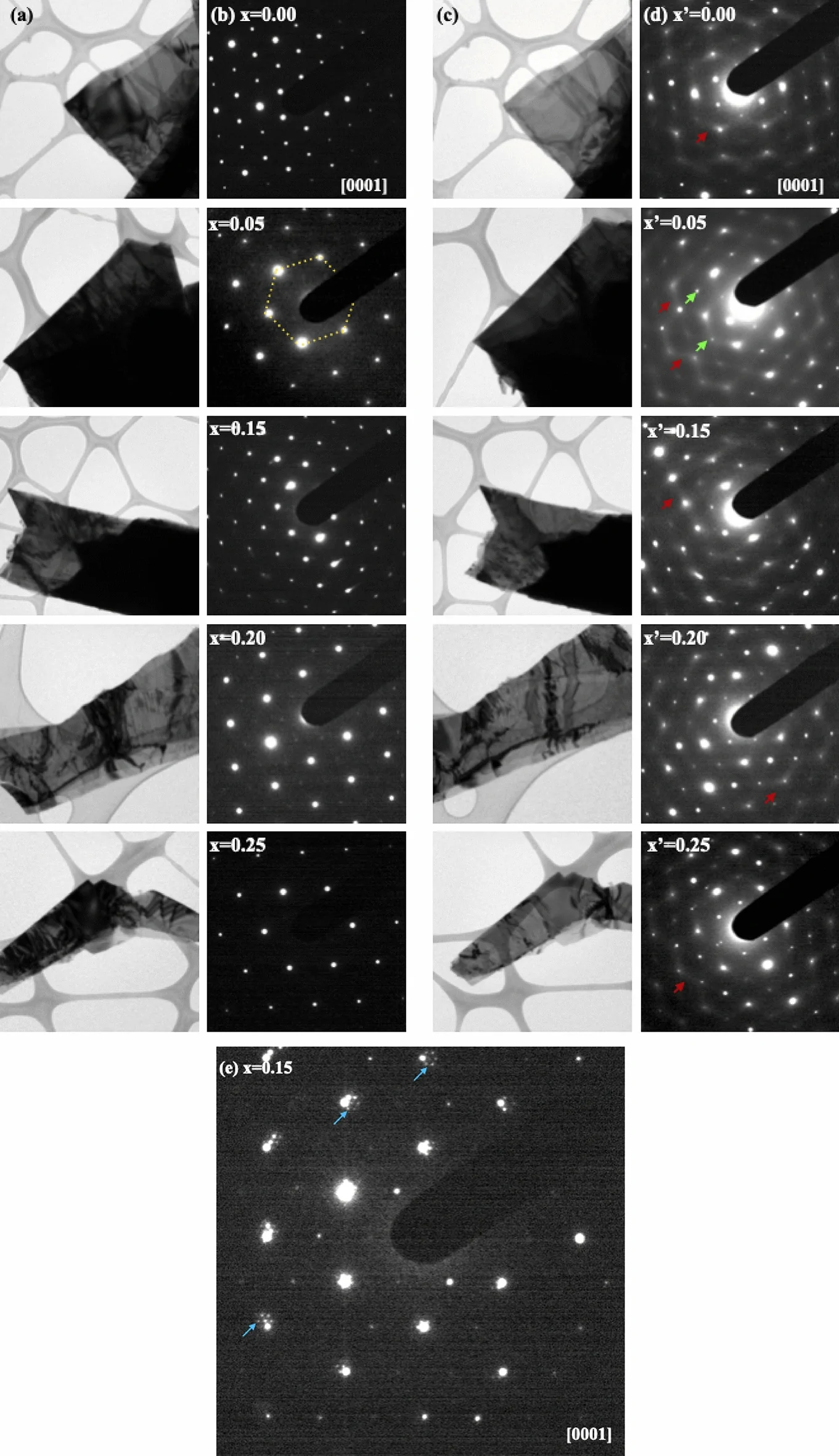
Structural characterization of Nb$\vphantom{0}_x$Bi$\vphantom{0}_2$Se$\vphantom{0}_3$. Room-temperature bright-field (BF) transmission electron microscopy (TEM) images and selected area electron diffraction (SAED) patterns for nominal concentrations *x = 0.00*, 0.05, 0.15, 0.20, and 0.25 (the *x = 0.00* data are from Ref.^30^). Columns: **(a, c)** BF-TEM images; **(b, d)** corresponding SAED patterns. Pair **(a,b)** are on the $\langle 0001\rangle$-zone axis; pair **(c,d)** are slightly off-axis ($<5^\circ$). **(e)** For *x = 0.15*, a $\langle 0001\rangle$-zone axis SAED pattern from a second sample region reveals satellite spots (blue arrows) evidencing a superlattice phase. The same camera length and magnification were used as in Ref.^31^; see that reference for scale calibration.

Doping of Nb atoms significantly modifies this structure (right panel of Fig. 1(a)). Like other foreign chemical dopants in Bi$\vphantom{0}_2$Se$\vphantom{0}_3$^14–16^, Nb preferentially occupies intercalation sites within the van der Waals gap, disrupting the original ABC stacking symmetry. Specifically, as depicted in the right panel of Fig. 1(b), Nb atoms (denoted as C’) insert into the structure, effectively changing the stacking order. This introduces a faulted sequence: -A(Se1)-B(Bi)-C(Se2)-A(Bi)-B(Se1)-**C’(Nb)**-A(Se1)-B(Bi)-C(Se2)-A(Bi)-. As a result, the periodic stacking faults manifest as a clear lattice distortion, which is identified in our selected area electron diffraction (SAED) patterns taken at room temperature in Fig. 2. At higher Nb doping concentrations (*x> 0.2*), Nb may substitute for Bi, introducing periodic stacking faults as also observed in previous research^31^.

Figure 2 presents electron diffraction analysis of doped Bi$\vphantom{0}_2$Se$\vphantom{0}_3$ samples (*x = 0.00*–0.25). Bright-field imaging (Fig. 2a) reveals a layered morphology with strong diffraction contrast, indicating high crystalline quality. Selected area electron diffraction (SAED) patterns along the $\langle 0001 \rangle$-zone axis (Fig. 2b) show fundamental Bragg reflections (e.g., highlighted yellow dots for *x = 0.05*). Across all doped Bi$\vphantom{0}_2$Se$\vphantom{0}_3$ samples studied, weaker $\frac{2}{3}$-order forbidden spots (indicated with green arrows) are observed, as shown for *x = 0.05*. The 2/3 diffraction spots correspond to the (100) and (010) crystal planes^31,32^. In a perfect ABC stacking sequence, these reflections are kinematically forbidden. Their appearance therefore indicates either a breakdown of the *c*-axis ABC stacking sequence symmetry or a superlattice reconstruction in the *ab*-plane that nevertheless retains the *c*-axis stacking order.

To probe the origin of these features, we tilted the crystals slightly ($<5^\circ$) off the zone axis. Under this condition, the $\frac{2}{3}$-order spots become pronounced and are present in all doped samples (Fig. 2c,d). Concurrently, diffuse streaks (e.g., highlighted red arrows in column Fig. 2(d)) appear between the Bragg spots. It is worth noting that we include the *x = 0.00* (nominally pristine) sample in this study, grown under the same conditions as the Nb-doped Bi$\vphantom{0}_2$Se$\vphantom{0}_3$ crystals. Bi/Se self-doping effects in the pristine sample produce similar structural features to those observed in the metal-intercalated samples, as previously reported for nominally pristine Bi$\vphantom{0}_2$Se$\vphantom{0}_3$^30^. In addition, on a separate *x = 0.15* crystal, we identified distinct satellite spots surrounding the primary reflections (Fig. 2e).

These diffraction features provide key structural insights. The ubiquity of these otherwise forbidden superlattice reflections at 2/3a* confirms that intercalation (whether from self-doping in pristine crystals or intentional Nb doping) induces local structural symmetry breaking and ABC stacking faults. Although the SAED patterns primarily provide in-plane diffraction information, the presence of these forbidden spots is a well-established indirect indicator of out-of-plane stacking faults. The diffuse streaks are a characteristic signature of a periodic lattice distortion^29,31,34–36^, while the satellite spots are consistent with the formation of superlattice structures in this system^29,32^. Similar observations have been reported for Sn, Fe, Co, and Cu-intercalated Bi$\vphantom{0}_2$Se$\vphantom{0}_3$^29,32^. The presence of these lattice and electronic instabilities prompted our investigation into long-range ordered states at lower temperatures.

**Fig. 3:**
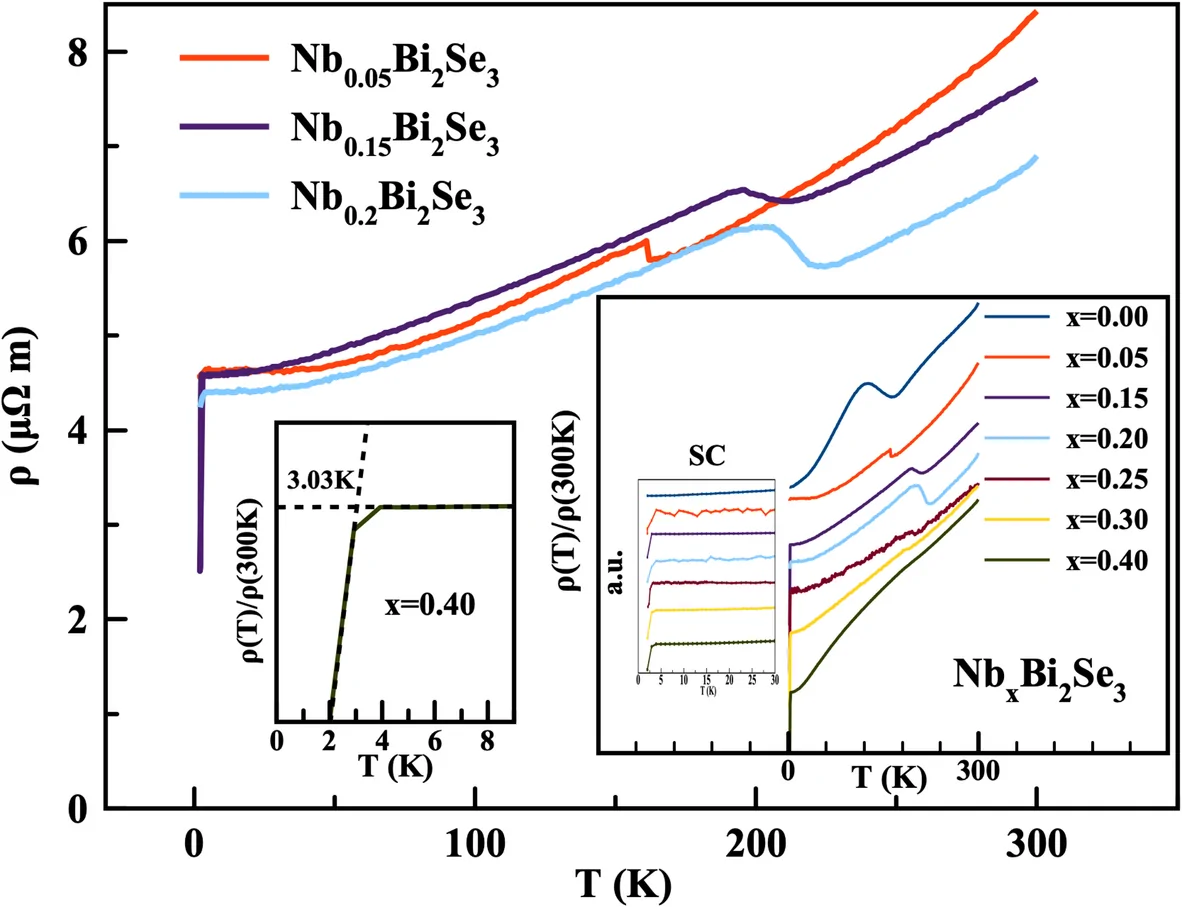
Electrical transport properties of Nb$\vphantom{0}_x$Bi$\vphantom{0}_2$Se$\vphantom{0}_3$. The main panel shows temperature-dependent resistivity *ρ (T)* for *x = 0.05*, 0.15, and 0.20 overlaid on a linear scale over the measured temperature range (1.8–300 K). The inset displays normalized resistivity $\rho (T)/\rho (300~\textrm{K})$ for *x = 0.00*–0.40 (the *x = 0.00* data are from Ref.^30^), with curves offset vertically for clarity and the superconducting transitions enlarged. A schematic on the left side of the inset illustrates the $T_c$ onset determination method (intersection of extrapolated normal-state and superconducting-state resistance lines).

## Emergent electronic and magnetic states

Resistivity measurements reveal a pronounced resistivity anomaly between 150 and 200 K in all Nb-intercalated samples (Fig. 3; the *x = 0.00* sample, included for reference, shows a similar transition arising from Bi/Se self-doping, with $T_{\text {anom}} \approx 140$ K). The transport data shows systematic shifts in this transition with nominal Nb content during crystal growth. The resistivity upturn is indicative of a partial gap opening near the Fermi level, though the system returns to metallic behavior below 140 K. This feature coincides with the emergence of both a superlattice state and of a periodic lattice distortion observed in electron diffraction (Fig. 2), where lattice and electronic instabilities are coupled. Taken together, these observations point to the opening of a partial Fermi-surface gap at $T_{\text {anom}}$ (determined from the minimum of *dρ /dT*, Supplementary Table 1). For compositions with *x> 0.00*, a sharp resistivity drop signifies the emergence of superconductivity below $T_c \approx 1.9$–3.5 K, consistent with previous reports^37^. The systematic shifts of both changes with doping level rule out an impurity origin.

**Fig. 4:**
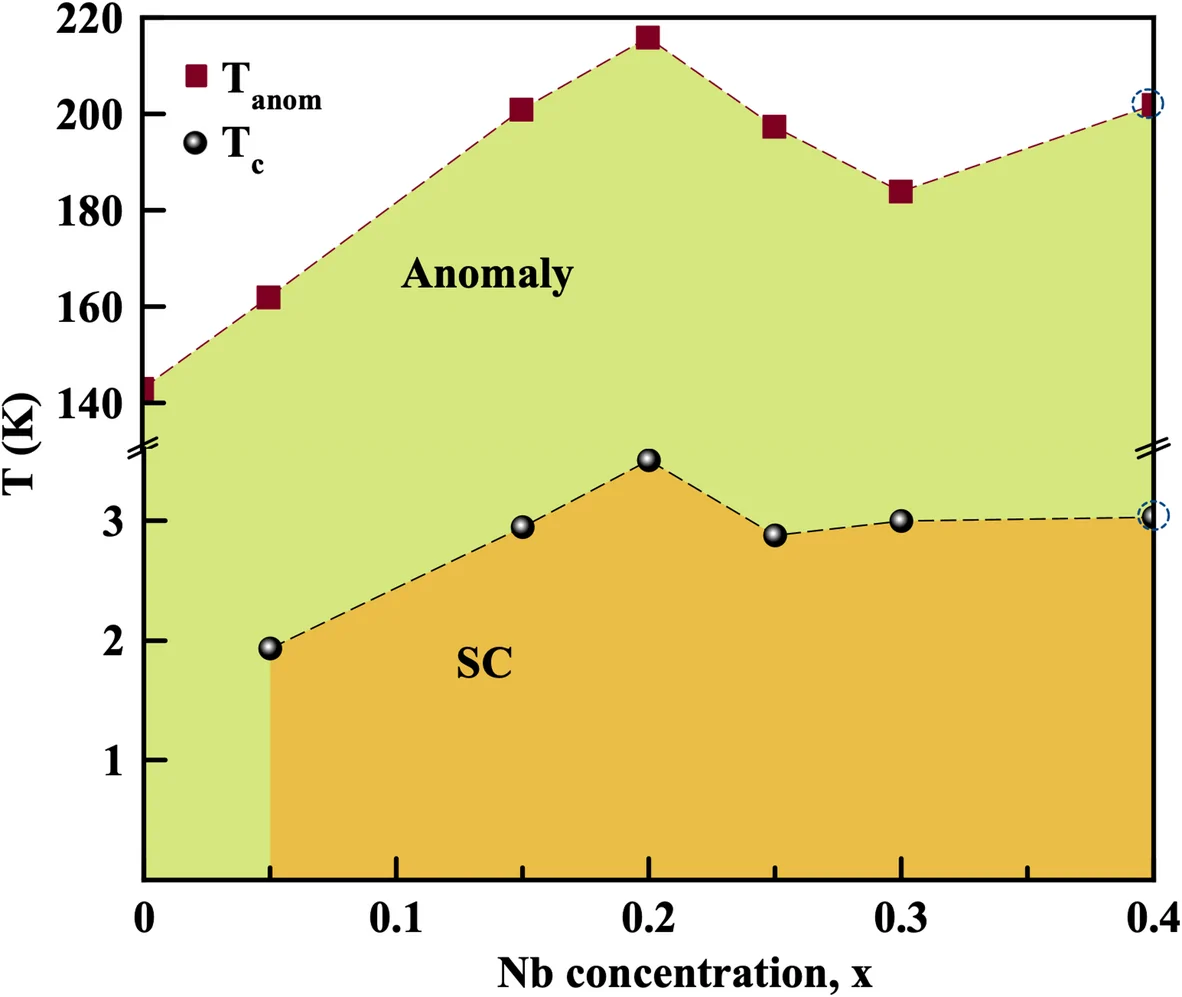
Qualitative phase diagram of Nb$\vphantom{0}_x$Bi$\vphantom{0}_2$Se$\vphantom{0}_3$. Transition temperatures vs. nominal Nb concentration *x*. $T_{\textrm{anom}}$ (red squares) from *dρ /dT* minimum; $T_c^{\textrm{onset}}$ (black circles) from extrapolated resistances. At *x = 0.40,* dashed blue circles mark two separate sample pieces: one showing only superconductivity, the other only the anomaly. For all other compositions, both features were observed in the same piece. Trends are discussed qualitatively; precise stoichiometry was not determined quantitatively.

The qualitative phase diagram in Fig. 4 maps the evolution of the anomalies and superconducting (SC) transition temperatures with Nb nominal concentration (*x*) in Nb$\vphantom{0}_x$Bi$\vphantom{0}_2$Se$\vphantom{0}_3$. In pristine samples (*x = 0*), the anomaly originates from self-doping by Bi or Se vacancies^30^. For $0.05 \le x \le 0.20$, the anomalies and SC were observed together, with both $T_c^{\textrm{onset}}$ and $T_{\textrm{anom}}$ increasing. This mutual enhancement stems from Nb intercalation and its associated structural disorder. At *x> 0.25*, the secondary phases from both BiNbSe$\vphantom{0}_3$ and BiSe start to increase with increasing Nb concentrations (Supplementary Fig. S1). These secondary phases create local strain fields that pin or suppress one electronic ground state in favor of the other, consistent with weakened slope changes in the transport anomaly. For example, at the highest doping (*x = 0.40*), separate SC and anomaly onsets were observed in different sample pieces (one in Fig. 3 with SC transition and the other in Supplementary Fig. S2 with anomaly changes), indicating competition between different electronic states, likely driven by impurity suppression.

**Fig. 5:**
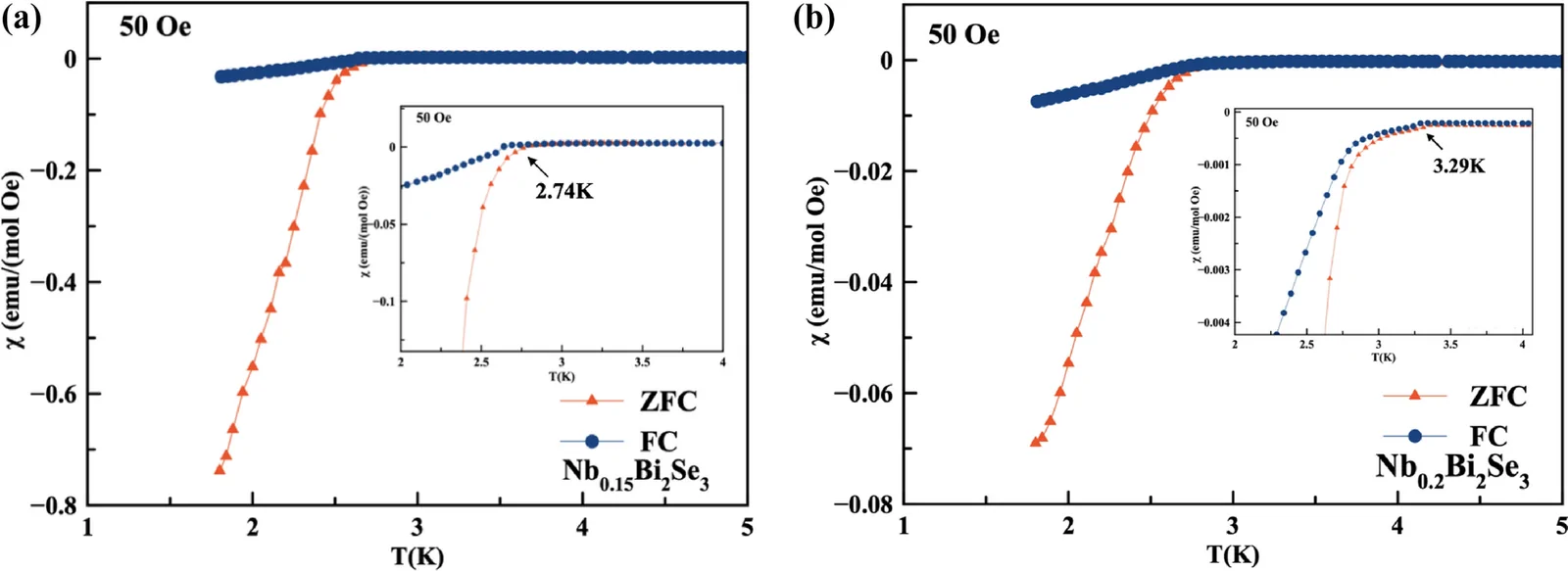
Temperature-dependent DC magnetic susceptibility of Nb$\vphantom{0}_{0.15}$Bi$\vphantom{0}_2$Se$\vphantom{0}_3$ and Nb$\vphantom{0}_{0.20}$Bi$\vphantom{0}_2$Se$\vphantom{0}_3$ samples. (**a**) Zero-field-cooled (ZFC) and field-cooled (FC) susceptibility for Nb$\vphantom{0}_{0.15}$Bi$\vphantom{0}_2$Se$\vphantom{0}_3$ measured in an applied field of *H = 50* Oe. (**b**) Corresponding ZFC and FC susceptibility for Nb$\vphantom{0}_{0.20}$Bi$\vphantom{0}_2$Se$\vphantom{0}_3$ under the same field. The insets show the determination of $T_c^{\textrm{onset}}$ from the ZFC susceptibility, yielding *∼2.74* K for *x = 0.15* and *∼ 3.29* K for *x = 0.20*.

Bulk superconductivity is confirmed by DC magnetic susceptibility measurements under a 50 Oe field applied parallel to the crystallographic *c*-axis (Fig. 5). For the composition *x = 0.15*, the zero-field-cooled (ZFC) susceptibility reveals a superconducting transition at $T_c^{\textrm{onset}} \approx 2.74$ K. Similarly, the *x = 0.20* crystal shows a transition at $T_c^{\textrm{onset}} \approx 3.29$ K. These results are consistent with the transition trend in Fig. 4.

To quantify the superconducting volume fraction and flux pinning, we compared the magnetization values at the lowest measured temperature (1.8 K) under ZFC and field-cooled (FC) conditions. The ratio of the absolute magnetization values, $|M_{\textrm{ZFC}} / M_{\textrm{FC}}|$, was used to assess the degree of flux trapping. The *x = 0.15* sample exhibits a $|M_{\textrm{ZFC}} / M_{\textrm{FC}}|$ ratio at 1.8 K that is approximately 2.5 times larger than that of the *x = 0.20* sample, indicating a lower concentration of secondary phases and, consequently, a higher superconducting volume fraction in the *x = 0.15* sample.

The superconducting transition temperature $T_c$ increases with Nb concentration *x* (with *x ≤ 0.2* in Fig. 4); thus, *x = 0.20* has a higher $T_c$ than *x = 0.15*. This suggests that Nb doping directly enhances the superconducting pairing strength or carrier density, even though higher doping levels also introduce more secondary phases. These two observations are consistent: higher Nb concentration raises $T_c$ while also introducing more secondary phases that reduce the superconducting volume fraction.

**Fig. 6:**
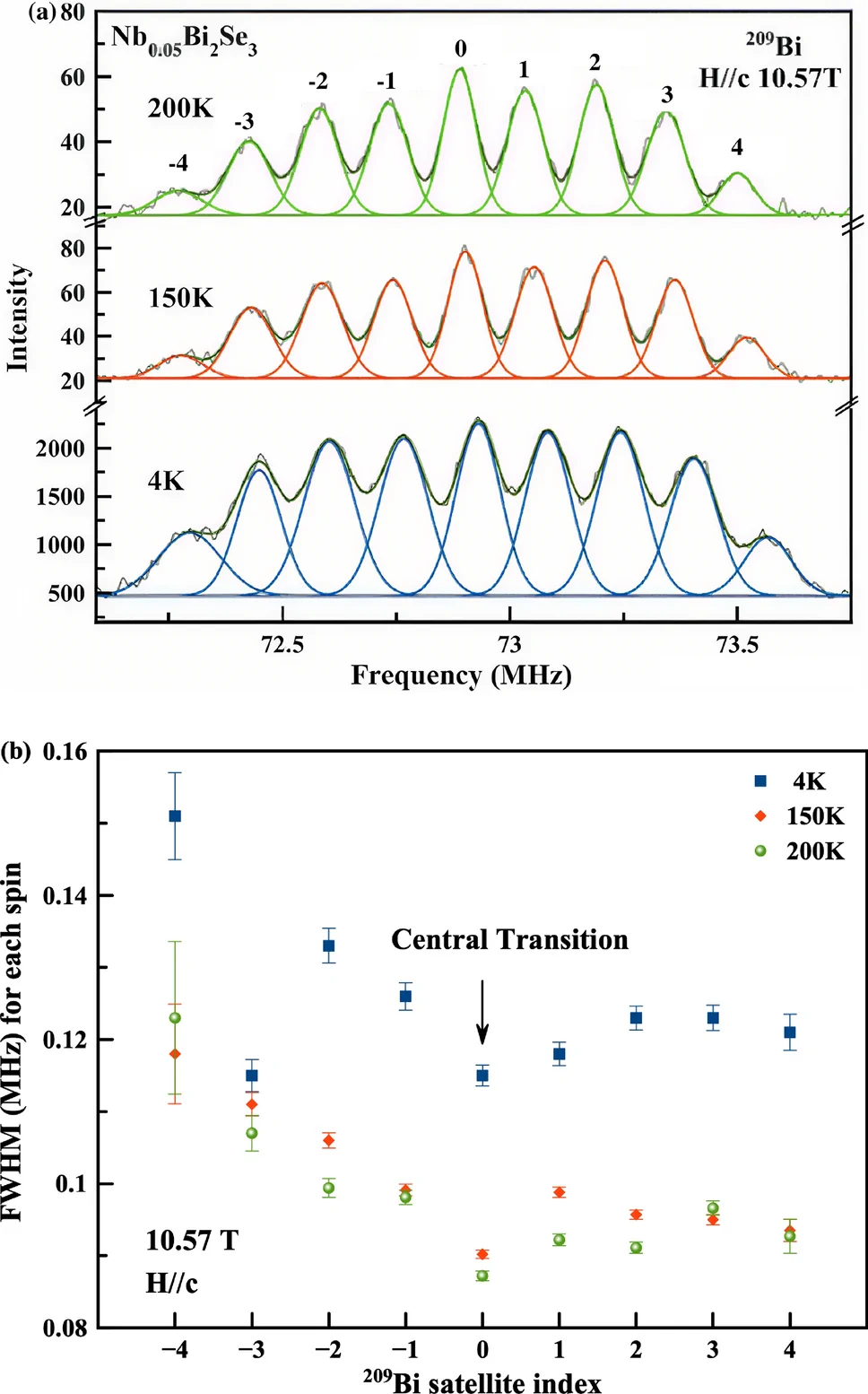
${}^{209}$Bi NMR in Nb$\vphantom{0}_{0.05}$Bi$\vphantom{0}_2$Se$\vphantom{0}_3$. (**a**) NMR spectra at *H = 10.57* T (*H ∥ c*) at temperatures spanning the anomalies ($T_{\textrm{anom}}$). The nine peaks are the first-order quadrupolar-split satellite transitions of the spin-*I=9/2* ${}^{209}$Bi nucleus, labeled by their satellite index from *-4* to *+4*. The central transition (*-1/2 ↔ +1/2*) is assigned the satellite index 0. (**b**) Full width at half maximum (FWHM) for each transition, extracted from fits in (a), vs. satellite index.

## Microscopic origin probed by NMR

Figure 6 displays $\vphantom{0}^{209}$Bi NMR measurements on a Nb$\vphantom{0}_{0.05}$Bi$\vphantom{0}_2$Se$\vphantom{0}_3$ crystal, performed with a static magnetic field of 10.57 T applied parallel to the crystal’s *c*-axis. This field strength is significantly above the superconducting upper critical field ($H_{c2} < \text{3 } \text{ T }$)^38^, ensuring the sample was in the normal state. Frequency-sweep spectra spanning 72–74 MHz were acquired at temperatures of 4 K, 150 K, and 200 K, corresponding to points below, near, and above the anomaly temperature ($T_{\text {anom}} =$161$\ \text {K}$). The characteristic nine-peak structure arises from the *I = 9/2* nuclear spin of $\vphantom{0}^{209}$Bi, and the peaks were fit with Gaussian functions to extract their linewidths.

The full-width at half-maximum (FWHM) for each peak is plotted as a function of satellite index in Fig.6(b). Upon cooling below $T_{\text {anom}}$, a pronounced broadening of approximately *30%* is observed for all but the third satellite transition, which remains an outlier. Furthermore, the satellite transitions display a progressive increase in linewidth and growing asymmetry with distance from the central transition. This specific pattern of broadening is characteristic of a distribution of local environments sensed by the Bi nuclei.

Although contributions from structural inhomogeneity cannot be entirely excluded, based on the complementary evidence from SAED, transport, and systematic doping studies, the observed broadening is consistent with a distribution of electric field gradients (EFGs) induced by the electronic anomaly state. The interaction of these site-specific EFGs with the ${}^{209}$Bi nuclear quadrupole moment is a well-established mechanism^39–41^. The observed broadening is consistent with a scenario where the electronic anomaly state creates a discommensurated structure—a state of incomplete or inhomogeneous order—leading to a distribution of EFGs, a well-established effect in canonical systems such as 2H-NbSe$\vphantom{0}_2$^39^ and others^40,41^.

## Discussion

Our work reveals a cooperative lattice and electronic instability in Nb-doped Bi$\vphantom{0}_2$Se$\vphantom{0}_3$, driven by doping-induced structural and electronic fluctuations. We trace this behavior to its origin in synthesis-controlled intercalation, demonstrating that the emergent electronic behavior is exquisitely sensitive to high-temperature quenching—a prerequisite for stabilizing the electronic anomaly state and the subsequent superconducting transition. The lattice symmetry breaking is unambiguously evidenced by periodic lattice distortions and superlattice reflections in SAED. Electronically, transport measurements reveal the opening of a partial gap followed by the onset of superconductivity, the latter confirmed by magnetic measurements. Furthermore, NMR line broadening indicates electric field redistribution consistent with the electronic anomaly, though contributions from structural inhomogeneity cannot be entirely excluded. These multidisciplinary findings suggest that the doping process itself—common to various species—produces structural and electronic modifications that simultaneously enable possible Fermi surface nesting and likely enhance electron-phonon coupling. This dual effect governs the observed behaviors through strong lattice-charge correlations, providing a fundamental link between electronic instabilities and superconductivity.

We pinpoint the origin of this sensitivity to quenching-controlled intercalation kinetics. The rapid thermal quench freezes a non-equilibrium configuration of dopants (Bi/Se self-doping or Nb intercalants) within the van der Waals gaps^18,30,31,42^, where dopants achieve two critical effects: they modulate the charge density, providing the carrier concentration necessary for both superconductivity and electronic anomaly formation, and they impose a distinct *c*-axis distortion on the host lattice^29,31,32^. While other structural defects may play a secondary role^20,21,23^, the dominant influence of quenching firmly identifies the chemical doping process itself as the key tuning parameter. This modifies both the crystal structure (creating stacking faults and lattice distortions) and the electronic structure (carrier density and Fermi level position), enabling the emergence of electronic instabilities and possible phase transitions. Together, these observations establish a synthesis-structure-property relationship, where targeted disorder engineering via dopants dictates the emergence of complex electronic behavior.

The importance of quenching is further supported by prior work. Our previous research on Cu-doped Bi$\vphantom{0}_2$Se$\vphantom{0}_3$ demonstrated that quenching is critical for stabilizing superconductivity; non-quenched samples exhibited no superconducting transitions^18^. This finding is consistent with Schneeloch et al.^42^, who also showed that quenching is required to induce and maintain the superconducting phase in doped Bi$\vphantom{0}_2$Se$\vphantom{0}_3$. Most directly, an in-situ X-ray diffraction study by Kevy et al.^43^ on Nb-doped Bi$\vphantom{0}_2$Se$\vphantom{0}_3$ found that non-quenched samples showed signs of NbSe$\vphantom{0}_2$ impurity phases, which were successfully removed by quenching. In contrast to quenched samples, which exhibit an electronic anomaly state and superconducting transition, non-quenched samples (based on the Cu analog and Kevy et al.^18,42,43^) are expected to show only metallic behavior without these features.

Having established the central role of quenching, we now examine the distinct structural and electronic consequences of doping within the ab-plane and along the *c*-axis. Our findings suggest that phase behaviors in doped topological insulators arise from dual dopant effects. In the *ab*-plane, dopants introduce stacking faults and electronic density modulations, producing the superlattice spots in our SAED data. Along the *c*-axis ($\it k_z$ direction), dopants modify the band structure, leading to strong Fermi nesting at small wave vectors *q*^20,21^, which could drive electronic anomaly along the *c*-axis. Whether this nesting influences the *ab*-plane superlattice is unclear; the *ab*-plane electronic density modulations may instead be confined to the planes. Consequently, the electronic anomaly observed in transport could reflect contributions from either or both phenomena. Future work should focus on confirming the precise mechanism.

Beyond the electronic anomaly, this instability may also be relevant to the emergence of topological superconductivity, as lattice distortions and possible Fermi nesting can enhance electron-phonon coupling. This microscopic picture implies that the presence or absence of topological superconductivity in a given material may be less about its nominal stoichiometry and more about the specific synthesis-controlled doping states that enable this underlying lattice instability. While dopant-specific effects (e.g., Nb *d*-orbitals) can modify the electronic structure^23^, the foundational physics governing the phase transitions is rooted in synthesis-controlled structural distortions. This insight provides a new materials-science-guided principle for the targeted design of topological superconductors.

Having discussed the possible mechanism for the observed phase behaviors, we now address and exclude the potential role of impurity phases as the source of the electronic anomaly and superconductivity.

We first exclude known Nb-based secondary phases as the origin of the electronic anomaly. The characteristic transition temperatures of NbSe$\vphantom{0}_2$ ($T_c = 7.2$ K, $T_{\textrm{CDW}} = 33$ K)^44^ and NbSe$\vphantom{0}_3$ ($T_{\textrm{CDW}} = 145$ K)^45^ are inconsistent with our data. Furthermore, the magnitude of the resistivity anomaly at $T_{\textrm{anom}}$ is too large to originate from a minor impurity phase. Most conclusively, the systematic evolution of $T_{\textrm{anom}}$ with Nb concentration (*x*)—including its presence in self-intercalated pristine Bi$\vphantom{0}_2$Se$\vphantom{0}_3$ and its systematic shift upon Nb doping—demonstrates its intrinsic nature within the Nb$\vphantom{0}_x$Bi$\vphantom{0}_2$Se$\vphantom{0}_3$ system.

Similarly, we establish the superconductivity as intrinsic. A minor impurity phase of fixed composition would exhibit a static $T_c$, whereas we observe a continuous, systematic increase of $T_c^{\textrm{onset}}$ from 1.9 K to 3.5 K with doping. Our XRD measurements show no evidence of known superconducting impurities such as Nb$\vphantom{0}_{0.9}$Bi$\vphantom{0}_{1.2}$Se$\vphantom{0}_3$^46^. While other secondary phases (e.g., BiSe) are detected at high *x> 0.25*, their appearance correlates with the suppression of the electronic anomaly and cannot explain the systematic emergence of superconductivity. Thus, the totality of the evidence indicates that both the electronic anomaly state and superconductivity are intrinsic properties of the material.

Taken together, these observations support the interpretation of the diffuse features and satellite spots as electronic modulations, consistent with similar reports in metal-doped Bi$\vphantom{0}_2$Se$\vphantom{0}_3$ systems^29–32^.

The relationship between the electronic anomaly and superconductivity (SC) in Nb$\vphantom{0}_x$Bi$\vphantom{0}_2$Se$\vphantom{0}_3$ is revealed to be of common origin rather than direct competition. At low doping (*x < 0.25*), the transition temperatures for both the anomaly and SC rise concurrently, driven by the increasing intercalation-induced *c*-axis distortion. This cooperative regime gives way to a simultaneous suppression of both states at higher doping (*x ≥ 0.25*), where the disruptive effects of scattering disorder dominate. This parallel response strongly suggests that the electronic anomaly and SC share a common structural origin and are susceptible to the same disordering forces.

A key question remains: does their coexistence represent a truly intertwined electronic state or mere spatial coexistence? Resolving this will require future low-temperature spectroscopic and scattering probes, such as neutron scattering and ARPES, to probe the mutual evolution of their order parameters below $T_c$. Ultimately, our work demonstrates that the phase behaviors in doped Bi$\vphantom{0}_2$Se$\vphantom{0}_3$ are governed by a synthesis-controlled lattice instability. To determine whether the electronic anomaly arises from charge ordering or structural effects (such as intercalation-induced structural disorder, stacking faults, frozen short-range periodic lattice distortions, or structural inhomogeneity), we propose future experiments such as temperature-dependent SAED or X-ray diffraction. To understand the correlations between the anomaly and superconducting states, low-temperature NMR measurements on similar compounds would also be valuable. This provides a foundational materials-science principle: the targeted engineering of dopant-induced disorder for manipulating complex electronic phenomena in topological quantum materials.

## Conclusion

In conclusion, we report the observation of a transport anomaly in Nb$\vphantom{0}_x$Bi$\vphantom{0}_2$Se$\vphantom{0}_3$ ($0.0 \le x \le 0.4$) and its coexistence with superconductivity in the doped samples (*x> 0*). This electronic anomaly emerges between 150 and 200 K (compared to *∼*140 K in pristine Bi$\vphantom{0}_2$Se$\vphantom{0}_3$), and is accompanied by a pronounced resistivity slope change, room-temperature superlattice reflections in SAED, and characteristic NMR line broadening below $T_{\textrm{anom}}$—three signatures that collectively point to simultaneous electronic and lattice instabilities. Strikingly, both $T_{\textrm{anom}}$ and $T_c$ exhibit nearly identical dome-shaped dependences on Nb content. Their concurrent rise at low doping and subsequent mutual suppression at higher *x* suggest a common structural sensitivity to intercalation-induced *c*-axis lattice distortion, rather than a simple competitive relationship. While candidates such as charge ordering or frozen lattice distortions remain under consideration, the microscopic origin of this anomaly is not yet fully resolved. Nevertheless, these results establish Nb$\vphantom{0}_x$Bi$\vphantom{0}_2$Se$\vphantom{0}_3$ as a promising platform for investigating the interplay between electronic and structural degrees of freedom on a topological background.

## Experimental methods

### Materials synthesis

Single crystals of Nb$\vphantom{0}_x$Bi$\vphantom{0}_2$Se$\vphantom{0}_3$ ($0 \le x \le 0.4$) were synthesized via a self-flux method. High-purity elemental powders of Bi (99.999%), Se (99.999%), and Nb (99.99%) were weighed in nominal ratios within an inert argon glove box to prevent oxidation. The 2.5 g mixtures were sealed under vacuum in high-quality quartz tubes. The ampoules were heated to 850 $\vphantom{0}^\circ$C, held for 20 hours for homogenization, and then slowly cooled to 650 $\vphantom{0}^\circ$C at a rate of 0.1 $\vphantom{0}^\circ$C/min. Finally, the samples were quenched in ice water to obtain the crystalline phase. Attempts to grow crystals with *x> 0.4* resulted in poor quality; therefore, these compositions are excluded from the present study.

### Structural characterization

Powder X-ray diffraction (XRD) patterns were collected on a Bruker D8 Discover diffractometer utilizing Cu K*α* radiation. The resulting patterns were analyzed via Rietveld refinement using the GSAS software with the EXPGUI interface. Selected Area Electron Diffraction (SAED) was conducted at 300$\,\textrm{kV}$ on a Hitachi H-9000NAR HRTEM. For these measurements, crystals of each composition (*x = 0.00*, 0.05, 0.15, 0.20, and 0.25) were gently ground in an agate mortar and pestle, and the powder was dispersed onto a lacey-carbon TEM grid. The same magnification (e.g., 1 nm scale bar) was used as in Ref. [31]; see that reference for scale calibration.

### Electrical and magnetic measurements

The electrical resistivity of single crystals was measured along the *c*-axis ($\textbf{J} \parallel c$) using a four-probe method in a Quantum Design Physical Property Measurement System (PPMS) with a ${9}\,\textrm{T}$ magnet. Electrical contacts were made using silver paste. Precise stoichiometry of Nb intercalation could not be determined due to random distribution of Nb atoms in the van der Waals gaps. Therefore, transport trends are reported versus nominal growth composition, and the results should be interpreted as qualitative trends rather than a quantitative phase diagram. DC magnetization measurements were performed on a Quantum Design Magnetic Property Measurement System (MPMS XL-5) equipped with a SQUID detector.

### Nuclear magnetic resonance (NMR)

^209^Bi NMR experiments were performed exclusively on a Nb_0.05_Bi_2_Se_3_ single crystal (dimensions: $\sim {5.3}\,\textrm{mm} \times {4.2}\,\textrm{mm} \times {1.2}\,\textrm{mm}$) using a pulsed NMR spectrometer. The crystal was aligned with the *c*-axis parallel to the external static magnetic field ($\textbf{H} \parallel c$, $H = {10.57}\,\textrm{T}$) in a home-built probe and cooled in an ${18}\,\textrm{T}$ cryostat. Spin-echo spectra were obtained by sweeping the frequency from ${72}\,\textrm{MHz}$ to ${74}\,\textrm{MHz}$ and processing the signals via the summed Fourier transform method.

## Supplementary Information

Supplementary Information.

## Data Availability

Data is available upon request.
