# Supplementary material for: Transport anomalies and lattice effects in superconducting Nb-doped Bi[image]Se[image]
Source: Sci Rep. 2026 Sep 15;16:28726. doi: 10.1038/s41598-026-63608-7 (PMC13578256; doi:10.1038/s41598-026-63608-7)
Supplement: Supplementary file 1 [file 41598_2026_63608_MOESM1_ESM.pdf]

Supplementary Materials

Transport Anomalies and Lattice Effects in Superconducting Nb-doped Bi_2Se_3

Yanan Li[a,b],[*] Christian Parsons[b], Anand Prashant Dwivedi[b], Nelson William [c,d], Ryan Baumbach [c,d], Marvin A. Schofield[b], Arneil P. Reyes[c,d], and Prasenjit Guptasarma[b]

[a] *College of Electronic and Information Engineering, Liaoning Technical University, 125105, Huludao, Liaoning, China.*

[b] *Department of Physics, University of Wisconsin-Milwaukee, 53211, Milwaukee, Wisconsin, USA*

[c] *National High Magnetic Field Laboratory, 32310, Tallahassee, Florida, USA and*

[d] *Florida State University, Tallahassee, 32310, Tallahassee, Florida, USA*

Table 1: Resistivity Anomaly temperature (T_{anom}) and superconducting transition temperature (T_c) versus Nb doping level x in $Nb_xBi_2Se_3$

Nb concentrations x	T_{anom}	T_{c}
0.00	142.95	/
0.05	161.94	1.93
0.15	200.94	2.95
0.20	215.90	3.50
0.25	197.39	2.88
0.30	183.93	3.00
0.40	201.92	3.02

* Corresponding author:liyanan@lntu.edu.cn

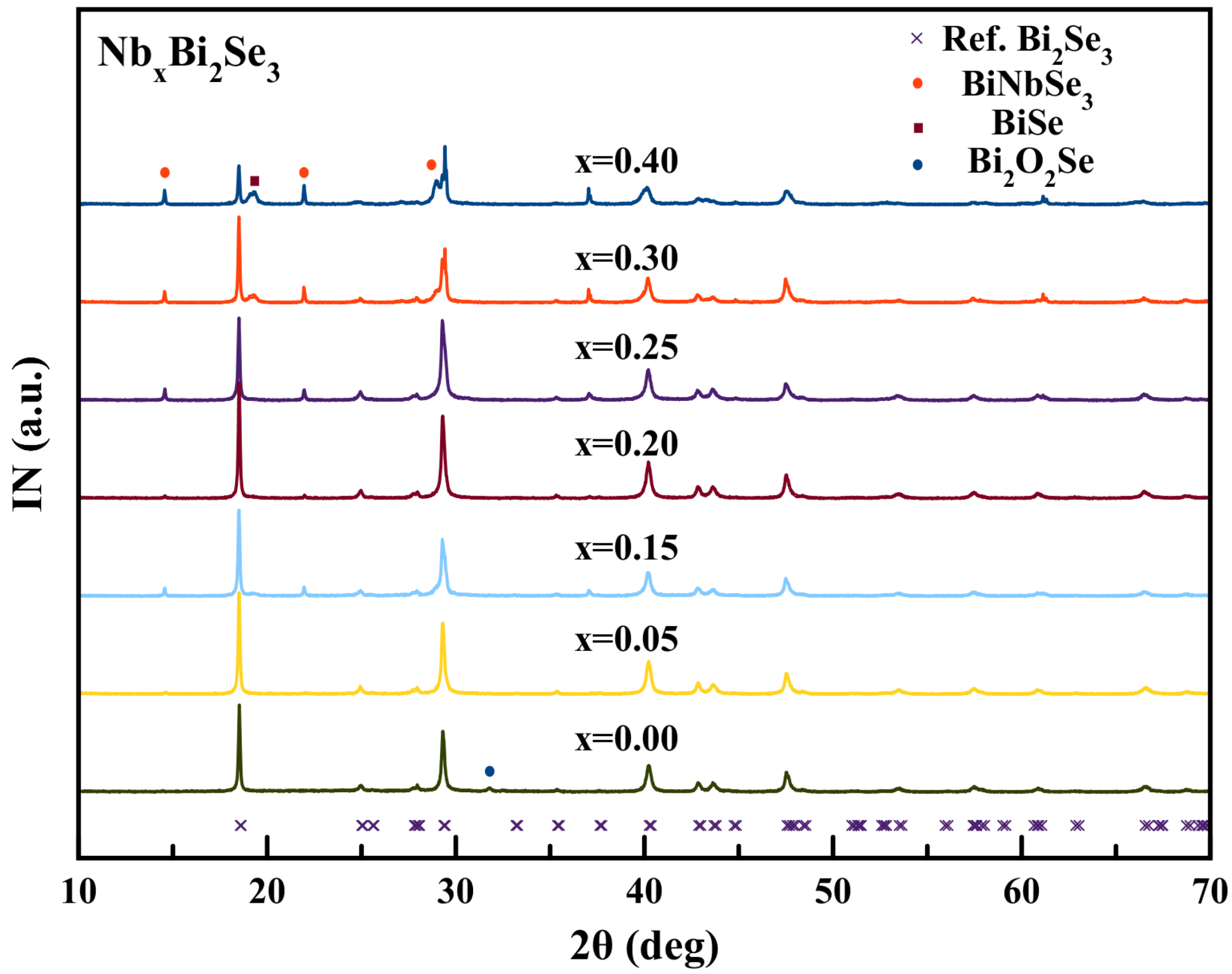

FIG. 1. Powder X-ray diffraction patterns of $Nb_xBi_2Se_3$ for $x = 0.00$, 0.05, 0.15, 0.20, 0.25, 0.30, and 0.40. Orange dots, red squares, and blue dots represent the secondary phases $BiNbSe_3$, BiSe, and Bi_2O_2Se, respectively. The presence of Bi_2O_2Se is due to grinding-induced oxidation.

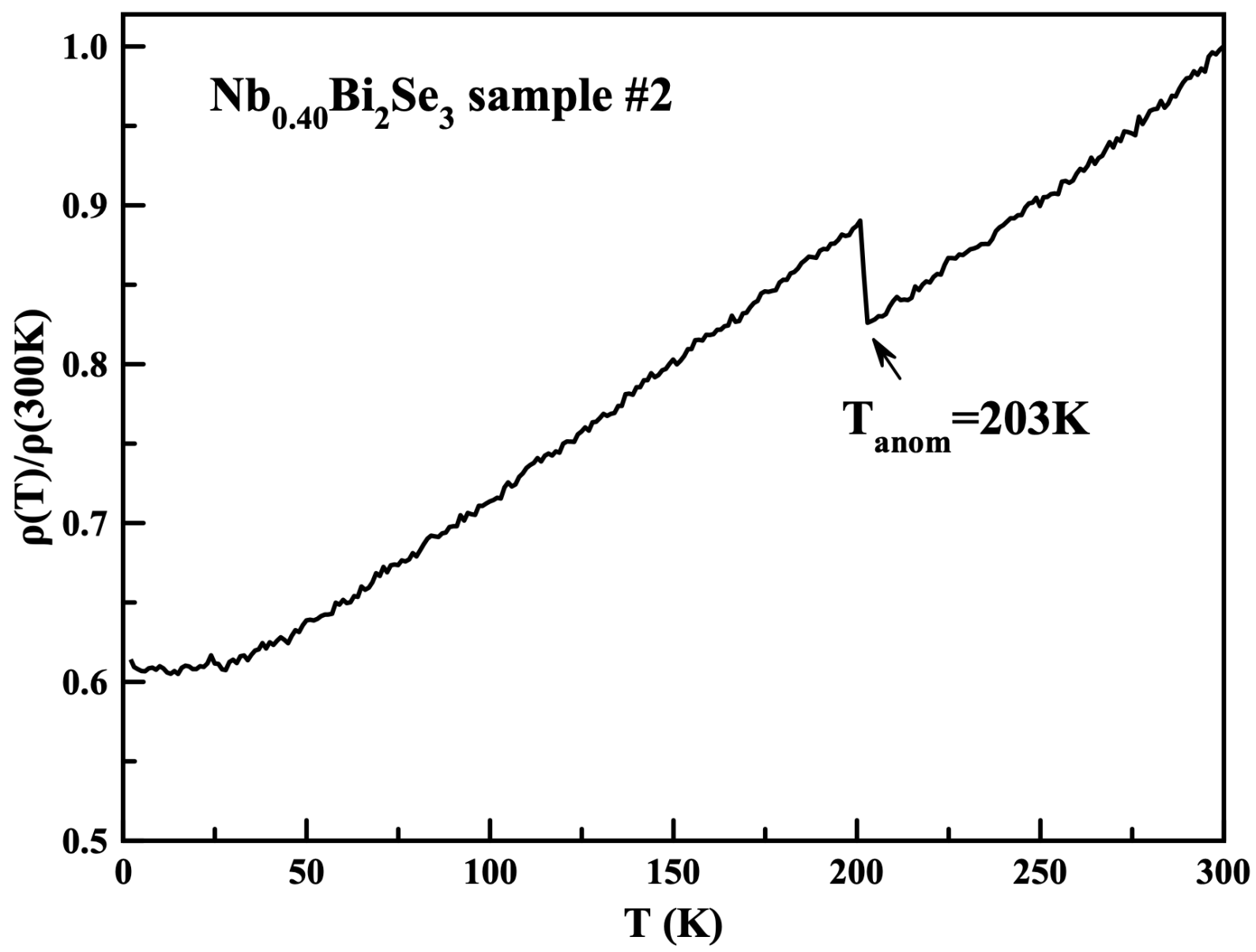

FIG. 2. Temperature-dependent $\rho(T)/\rho(300\ \mathrm{K})$ for a second sample of $\mathrm{Nb_{0.40}Bi_2Se_3}$ shows the resistivity anomaly temperature $T_{\mathrm{anom}} = 203$ K; no superconducting component is observed.
